# No bidirectional relationship between depression and periodontitis: A genetic correlation and Mendelian randomization study

**DOI:** 10.3389/fimmu.2022.918404

**Published:** 2022-07-22

**Authors:** Michael Nolde, Birte Holtfreter, Thomas Kocher, Zoheir Alayash, Stefan Lars Reckelkamm, Benjamin Ehmke, Hansjörg Baurecht, Sebastian-Edgar Baumeister

**Affiliations:** ^1^ Institute of Health Services Research in Dentistry, University of Münster, Münster, Germany; ^2^ Department of Restorative Dentistry, Periodontology, Endodontology, and Preventive and Pediatric Dentistry, University Medicine Greifswald, Greifswald, Germany; ^3^ Clinic for Periodontology and Conservative Dentistry, University of Münster, Münster, Germany; ^4^ Department of Epidemiology and Preventive Medicine, University of Regensburg, Regensburg, Germany

**Keywords:** periodontitis, mendelian randomization analysis, depression, genetic correlation, genetic correlation analysis

## Abstract

**Background:**

Observational and *in-vivo* research suggested a bidirectional relationship between depression and periodontitis. We estimated the genetic correlation and examined directionality of causation.

**Methods:**

The study used summary statistics from published genome wide association studies, with sample sizes ranging from 45,563 to 797,563 individuals of European ancestry. We performed linkage disequilibrium score regression (LDSC) to estimate global correlation and used Heritability Estimation from Summary Statistics (ρ-HESS) to further examine local genetic correlation. Latent Heritable Confounder Mendelian randomization (LHC-MR), Causal Analysis using Summary Effect estimates (CAUSE), and conventional MR approaches assessed bidirectional causation.

**Results:**

LDSC observed only weak genetic correlation (r_g_ = 0.06, P-Value = 0.619) between depression and periodontitis. Analysis of local genetic correlation using ρ-HESS did not reveal loci of significant local genetic covariance. LHC-MR, CAUSE and conventional MR models provided no support for bidirectional causation between depression and periodontitis, with odds ratios ranging from 1.00 to 1.06 in either direction.

**Conclusions:**

Results do not support shared heritability or a causal connection between depression and periodontitis.

## Introduction

Major depressive disorder and periodontitis are very common diseases (1, 2). Depression increases the risk of developing a wide range of medical disorders later in life (3). The association between depression and periodontitis has received attention both in the observational and *in-vivo* literature, suggesting that depression increases periodontitis risk and vice versa (4–8). A complex interplay of biological, cognitive, and behavioral mechanisms could account for the potentially bidirectional association. Intriguingly, the genetic susceptibility for depression is highly correlated with the polygenic risk for systemic inflammation, providing a putative biological pathway for the association with periodontal disease (9). An *in-vivo* experimental study demonstrated an inflammation-mediation causal mechanism of periodontitis on depression-like behavior in mice (10). This finding provides a translational link to a human genetic correlation study that indicated overlap between oral and mood conditions (11). It seems plausible that systemic inflammation represents a pathogenetic factor for depression and periodontitis, as animal models and experimental human studies have demonstrated that inflammatory signals can induce depression and periodontitis (12–16). Such inflammatory signals from the periphery interact with neurotransmitters (such as serotonin *via* the kynurenine pathway), alter neuroplasticity, and promote excitotoxicity and oxidative stress (8, 16, 17). Increased activation and impaired feedback regulation of stress response systems such as the hypothalamic-pituitary-adrenal axis and the sympathetic-adrenal-medullary system are the best validated biomarkers for depression (18). Similarly, some evidence suggests that increased urinary noradrenaline and cortisol levels are associated with periodontitis phenotypes (8, 19). Thus, the link between depression and periodontitis is likely to be mediated by salivary and blood cortisol, liposaccharides and other markers of cellular and systemic stress inflammation (20). Psychological stress causes immune responses that increase susceptibility to infection and potentially to the onset and progression of periodontal disease. Increased immune-inflammatory markers such as proinflammatory cytokines, oxidative and nitrosative stress markers, neurotoxic metabolites of tryptophan degradation and reduced neurotrophic levels co-occur with depression. A meta-analysis revealed elevated levels of interleukin (IL) 6, tumor necrosis factor (TNF) alpha, TNF receptor 2, IL-10, IL-2 receptor, C-C motif chemokine ligand 2, IL-13, IL-18, IL-12, IL-1 receptor antagonist and reduced interferon gamma in patients with depression (12). Similarly, members of the IL-1, IL-6, IL-10, IL-12, TNF families, and interferon gamma are also involved in the etiology of periodontitis (21, 22). Periodontitis originates from dysbiosis of the oral microbiome initiated by keystone pathogens (predominantly *Porphyromonas gingivalis*) that light up inflammatory response through release of liposaccharides and downregulation of neutrophil recruitment (23). This local host immune activation leads to tissue destruction and exaggerated osteoclastic activity and systemic immune dysregulation. These signals are postulated to spread through humoral, cellular and neural routes and reach the brain where neuroinflammation is initiated (20, 24). A growing literature further supports a role for alterations in the brain-gut microbiome axis in the etiology of depression (25, 26). A potential mechanism connecting periodontitis and depression is translocation of periodontal pathogens to the brain. In experimental mouse models, leaky mouth processes were demonstrated for *Fusobacterium nucleatum* and *P. gingivalis* (10, 20, 27).

A cognitive model for depression maps the etiology of depression within a diathesis-stress model and can also be used to attribute comorbid depression in oral disorders. Medical (including oral) conditions pose manifold stressors. If depression is viewed with a stress-coping model, depression in physical illness may occur when the demands exceed the coping resources (8, 28). A key adaptive task is to maintain a state of emotional equilibrium and manage the ongoing load of chronic stressors. The inability to achieve this task may lead to depression through a range of cognitive, behavioral, and social factors. Lifestyles such as unhealthy diet, smoking and heavy alcohol consumption are risk factors for depression and periodontitis (3, 29). These and other behaviors (including difficulties in access to dental care and lack of adherence to dental treatment and oral hygiene) could, in part, mediate the influence of depression on periodontitis (8, 30).

However, there are caveats to the conclusion that inflammatory, immune-modulated, and behavioral pathways underlie the relationship between depression and periodontitis. First, regarding the behavioral mediators, convincing experimental studies showing that reductions in unhealthy behaviors break the link between depression and periodontitis have not been conducted. Such studies are difficult to conduct as individuals cannot be randomly assigned to behaviors and because effects on periodontal parameters would take several years to emerge. Second, several of the available prospective observational studies on depression and periodontitis included lifestyle factors and inflammatory markers as covariates (7), and these studies found that associations persisted after covariate adjustment, casting doubt on the causal processes inferred from these studies.

In this study, we leveraged genome wide association study (GWAS) summary statistics for depression and periodontitis to investigate the shared genetic background between the traits. We quantified genome wide genetic correlation and performed bidirectional Mendelian randomization (MR) analyses to disentangle the putative direction of causation between depression and periodontitis.

## Materials and methods

### Overall study design

We used linkage disequilibrium score regression (LDSC) to estimate single-trait heritability and the shared genetic overlap between traits (31). LDSC allows assessment of phenotype heritability and co-heritability between traits based on single nucleotide polymorphisms (SNPs). A latent causal variable (LCV) model examined genetic causation (32). Current MR methods that accommodate correlated pleiotropy tested bidirectional causation (33, 34). MR enables evaluation of potential bidirectional causal association between traits based on the Mendel law that genetic variants are inherited independently, providing a natural analog to a randomized controlled trial (RCT) (35). The study was reported based on recommendations by STROBE-MR and ‘Guidelines for performing Mendelian randomization investigations’ (36, 37). The study protocol and details were not preregistered.

### Data sources

Genetic association estimates of SNPs with depression were obtained from the largest published European-ancestry GWAS meta-analysis to date (38), which included UK Biobank (127,552 cases; 233,763 controls), 23andMe (75,607 cases; 231,747 controls), and Psychiatric Genomics Consortium (43,204 cases; 95;690 controls) (**Table 1**). Depression was defined based on responses to web-based surveys, structured diagnostic interviews, or electronic medical records, with individuals who self-reported as having received a clinical diagnosis of or treatment for depression. GWAS analyses adjusted for age, sex, genotyping platform, and principal components. Genetic associations for periodontitis were obtained from GWAS of European studies contributing to the GeneLifestyle Interactions in Dental Endpoints (GLIDE) consortium (39), totaling 17,353 periodontitis cases and 28,210 controls. Periodontitis cases were classified by either the Centers for Disease and Control and Prevention/American Academy of Periodontology (CDC/AAP) or the Community Periodontal Index (CPI) case definition or through study participant reports of diagnosis of periodontitis. GWAS using the most recent classification of periodontitis (40) are not available yet. GWAS accounted for age, sex, and principal components.

**Table 1:** Description of genome wide association studies used for each phenotype.

Phenotype	Cases	Controls	Sex	PMID
Depression	246,363	551,200	49% female	30718901
Periodontitis	17,353	28,210	54% female	31235808

PMID, PubMed identifier.

### Statistical analysis

We estimated liability-scale heritability (*h²*) and genetic correlation (*r_g_*) of depression and periodontitis using LDSC (31). Summary statistics were filtered according to HapMap3. SNPs were excluded if they were strand-ambiguous or had a minor allele frequency of <0.01. Pre-computed LD scores and weights for the European population based on 1000 Genomes were used [https://alkesgroup.broadinstitute.org/LDSCORE/]. Univariate LDSC was performed to estimate *h²*, assuming the sample and population prevalence at 38% (38) and 10% (1) for depression, and 35% (39) and 16% (2) for periodontitis, respectively. Bivariate LDSC estimated *r_g_* for the genetic correlation between depression and periodontitis. LCV assumes that the genetic correlation between two traits is mediated by a latent variable and distinguishes causality from uncorrelated and correlated pleiotropy by estimating the genetic causality proportion using all genetic variants (32). LCV accounts for the genetic correlation between the two traits using cross-trait genetic correlations estimated from LDSC. The genetic causality proportion reflects the relative proportions of heritability of each trait that are explained by a shared latent factor, with higher magnitude estimates suggesting a causal effect and lower magnitude estimates suggesting correlated pleiotropy. We used ρ-HESS (41) as an alternative approach to estimate heritability and global correlation, and to further analyze local genetic correlation (42), dividing the genome into 1,703 approximately independent linkage disequilibrium (LD)-regions (43).

Standard MR approaches assume that SNPs robustly associate with the exposure (relevance), do not share common causes with the outcome (exchangeability), and affect the outcome exclusively through its effect on the exposure (exclusion restriction) (35). In most conventional polygenic MR methods ‘exclusion restriction’ is replaced by a weaker InSIDE (instrument strength independent of direct effect) assumption, which requires that instrument strength is uncorrelated to the direct (horizontal pleiotropic) effect on outcome and direct effects are on average zero (44). Violations of the InSIDE assumption can occur if several genetic variants relate to confounders of the exposure-outcome relationship. Latent Heritable Confounder MR (LHC-MR) (33) incorporates a latent confounder and models its contribution to exposure and outcome, while simultaneously estimating bidirectional effects of the two traits. These features make LHC-MR suitable for our purpose because we assume (1) that depression and periodontitis share common causes (i.e., liability, inflammation, microbiota, cognition, behavior) and (2) that traits mutually affect each other. LHC-MR links genome-wide associations with traits and confounder using a structural equation model. Like LHC-MR, Causal Analysis using Summary Effect estimates (CAUSE) (34) increases detection power by leveraging on more approximately independent SNPs. CAUSE is robust to InSIDE violations by allowing a proportion of variants to have direct pleiotropic effects mediated through a modeled latent factor. Conventional bidirectional MR models (multiplicative random-effects inverse-variance weighted (IVW), penalized weighted median, radial regression, and MR-pleiotropy residual sum and outlier (MR-PRESSO)) were included as sensitivity analysis (35, 44, 45). Given the few instrumental SNPs for periodontitis, we limited reporting for the effect of periodontitis on depression to the IVW and penalized weighted median methods. For conventional MR models, we selected 100 SNPs for depression using a GWAS P-value <5x10^-8^ and linkage disequilibrium r²<0.01 across a 1 Mb window (**Supplementary Table S1**). The SNPs explained 9.4% of the variability of depression and the minimum F statistic was 30.2. For periodontitis, we selected 8 SNPs using a GWAS P-value <5x10^-6^ and linkage disequilibrium r²<0.01 across a 1 Mb window (**Supplementary Table S2**). The SNPs explained 0.04% of the phenotypic variance and the minimum F statistic was 21.0. Odds ratios (ORs) were scaled to a doubling in exposure prevalence (46). *A priori* statistical power was calculated according to Brion et al. (47), which exploits asymptotic theory and derives the power *via* the non-centrality parameter from the respective asymptotic *χ* ^2^-distribution. On a 5% significance level, our analyses had a power of >85% to detect a causal effect of OR = 1.1 of depression on periodontitis and a power of >80% for a causal effect of OR = 1.5 of periodontitis on depression. We assessed heterogeneity using Cochran Q, I_GX_^2^, and performed the MR-PRESSO outlier test (48, 49). The MR Egger intercept test was used to test for directional pleiotropy (49). We performed most analyses using R version 4.1.2 (R Foundation for Statistical Computing) in combination with the cause, GenomicSEM, LCV, lhcMR, MRPRESSO, and TwoSampleMR packages. For analyses of local genetic correlation, we used the HESS software package version 0.5.3-beta under python 2.7.18 and the PySnpTools module in version 0.3.14.

## Results

**Figure 1:**
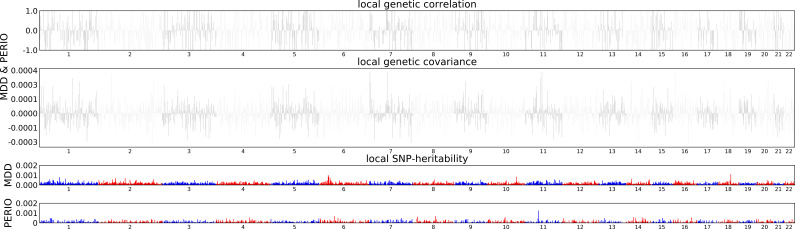
Local genetic covariance estimates.

**Figure 2:**
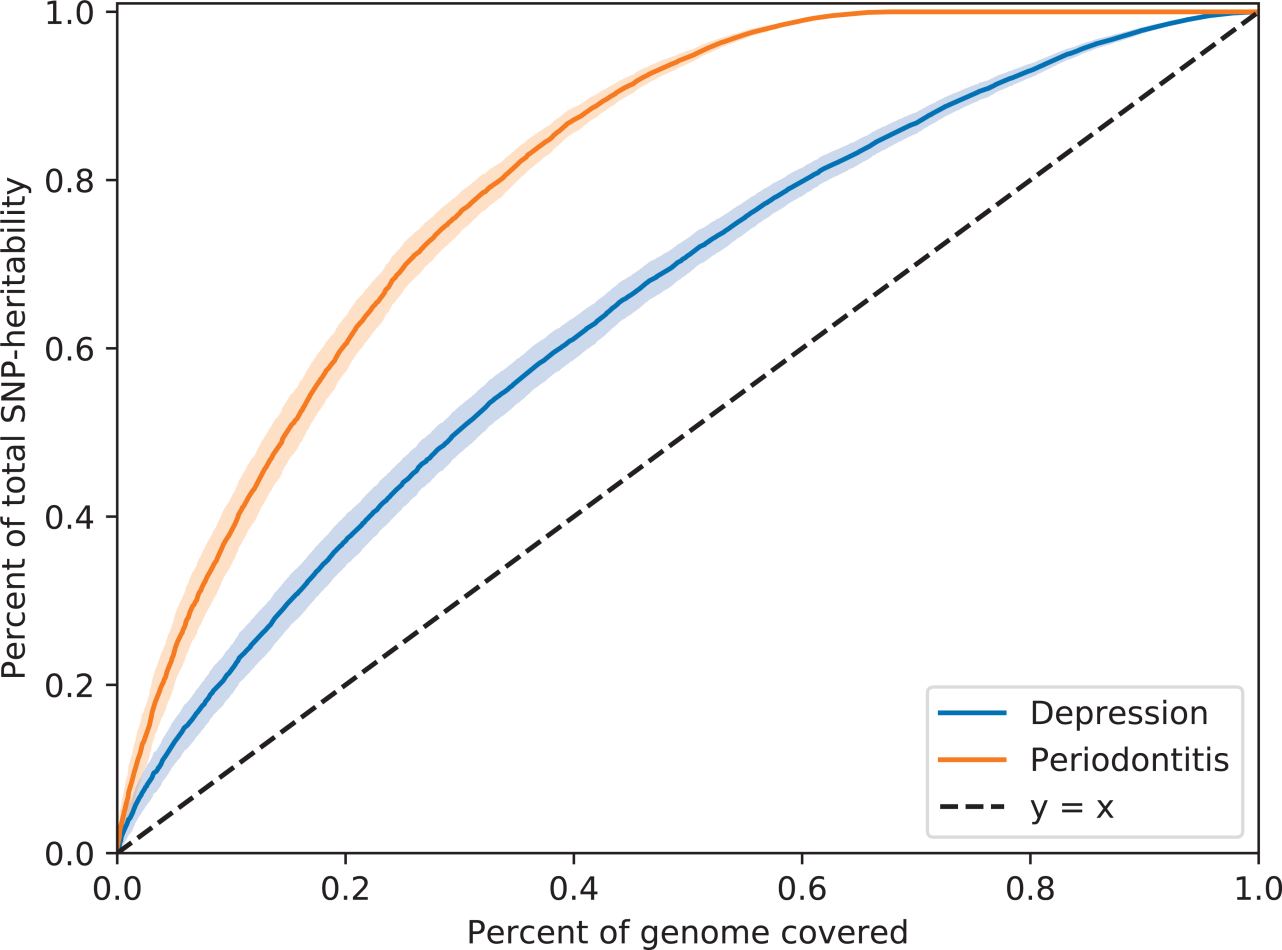
Contrasting polygenicity between depression and periodontitis.

**Figure 3:**
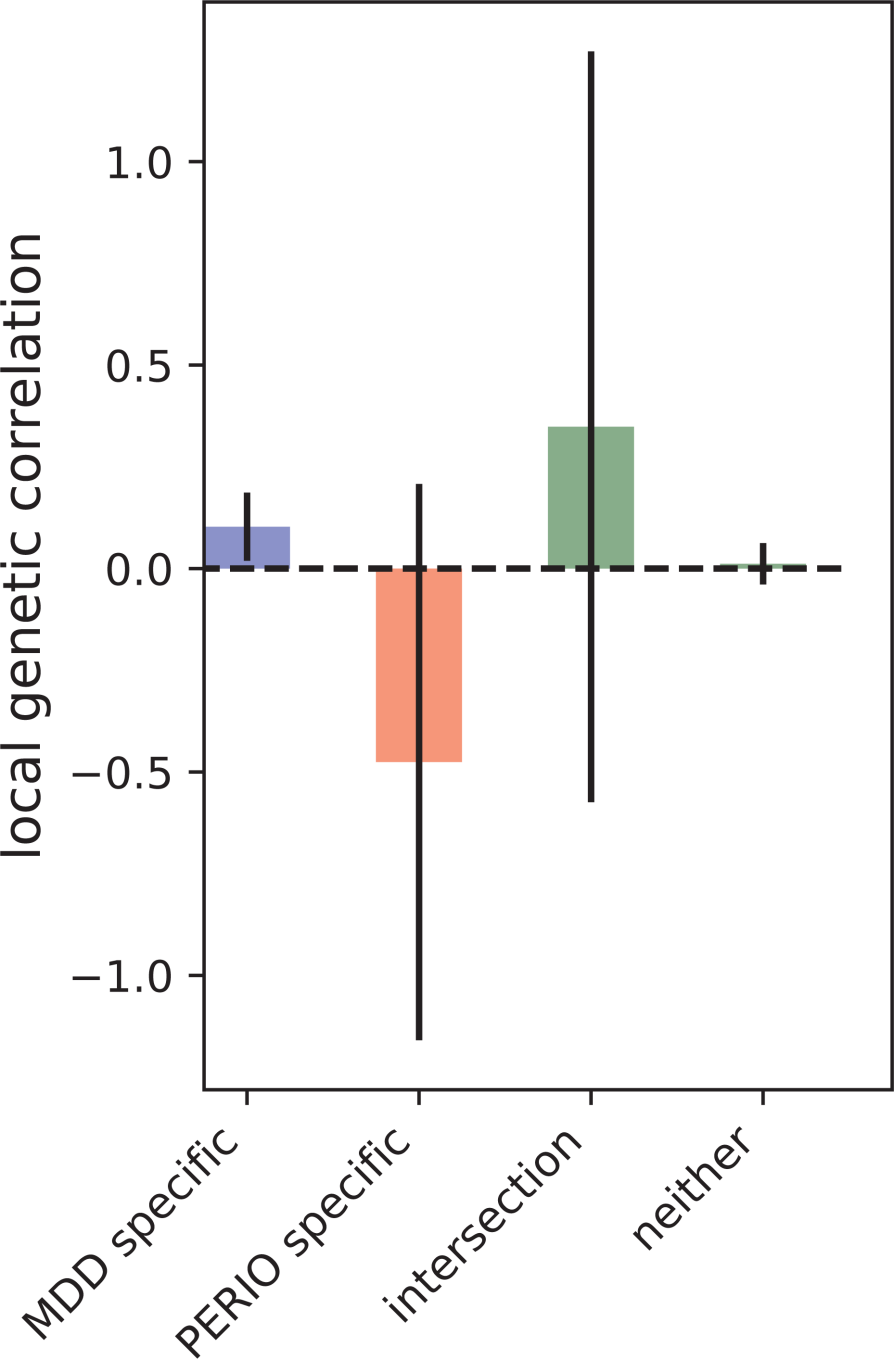
Local genetic correlation at loci ascertained for GWAS hits (p<5e-6) specific to depression and periodontitis.

SNP-based liability-scale heritability *h²* for depression and periodontitis were 19.6% (standard error (SE) = 0.8%) and 1.4% (SE = 1.2%) when estimated using LDSC, as well as 31% (SE = 0.6%) and 12.7% (SE = 2.3%) using ρ-HESS respectively (**Table 2**). We then performed bivariate LDSC and identified a weak genetic correlation between depression and periodontitis (r_g_ = 0.06, P-value = 0.619). Analysis of local genetic correlation did not reveal loci of significant local genetic covariance (**Figure 1**). Polygenicity was higher for depression than for periodontitis (**Figure 2**). Still, this might be due to the smaller size of the GWAS for periodontitis, rather than owed to biology. Inference for putative causality, represented by the local correlation in regions, where both traits showed significant SNPs (labelled ‘intersection’) was not significant but yielded point estimates compatible with shared genetic influences (**Figure 3**). Application of LCV provided an estimated genetic causality proportion of 0.42 (standard error SE = 0.26, P-Value for the null hypothesis of no partial genetic causality P_LCV_ = 0.300).

**Table 2:** Heritability of depression and periodontitis and their genetic correlation estimated using LDSC and ρ-HESS.

		Method	Depression	Periodontitis
Single-trait	Heritability h² (SE)	LDSC	0.196 (0.008)	0.014 (0.012)
Single-trait	Heritability h² (SE)	ρ-HESS	0.31 (0.00551)	0.127 (0.0227)
Cross-trait	Genetic correlation r_g_ (SE), P-value	LDSC	0.063 (0.127), 0.619	
Cross-trait	Genetic correlation r_g_ (SE), P-value	ρ-HESS	0.036 (0.022), 0.279	

LDSC, linkage disequilibrium score regression. ρ-HESS, Heritability Estimation from Summary Statistics. SE, standard error.

LHC-MR and CAUSE provided no evidence for a putative causal effect of depression on periodontitis (**Table 3**). In reverse, there was no evidence for an effect of periodontitis on depression. Estimates from LHC-MR and CAUSE were supported in conventional bidirectional MR analyses (**Table 4**). ORs for the effect of depression on periodontitis ranged from 1.02 to 1.06. ORs for the effect of periodontitis on depression ranged from 1.00 to 1.04. There was no heterogeneity in the IVW analyses (**Supplementary Table S3**). The intercepts from the MR Egger regression were centered around zero and provided no evidence for unbalanced pleiotropy (**Supplementary Table S3**). Using the MR-PRESSO global test, we found no evidence for outliers (*p*-value for the analysis of the effect of depression on periodontitis = 0.59; *p*-value the analysis of the effect of periodontitis on depression = 0.19)

**Table 3:** Estimates from LHC-MR and CAUSE for the bidirectional association of depression and periodontitis.

Direction	Method	OR^a^	(95% CI)	P-value
Depression ➔ Periodontitis	LHC-MR	1.06	(0.97; 1.16)	0.168
	CAUSE	1.05	(0.93; 1.19)	0.633
Periodontitis ➔ Depression	LHC-MR	1.04	(0.84; 1.29)	0.694
	CAUSE	1.00	(0.70; 1.42)	0.960

CAUSE, Causal Analysis Using Summary Effect estimates, LHC-MR, Latent Heritable Confounder MR.

CI: Confidence Interval for LHC-MR or Bayesian Credible Interval for CAUSE.

a OR (odds ratio) per doubling in exposure prevalence.

**Table 4:** Conventional MR methods for the bidirectional association of depression and periodontitis.

Direction	Method	OR^a^	(95% CI)	P-value
Depression ➔ Periodontitis	IVW	1.05	(0.95;1.15)	0.354
	Penalized weighted median	1.02	(0.89;1.16)	0.817
	IVW radial	1.05	(0.95;1.15)	0.354
	MR PRESSO	1.05	(0.95;1.15)	0.356
Periodontitis ➔ Depression	IVW	1.02	(0.99;1.04)	0.193
	Penalized weighted median	1.01	(0.99;1.03)	0.347

IVW, multiplicative random-effects inverse-variance weighted model. MR PRESSO, MR Pleiotropy RESidual Sum and Outlier.

a OR (odds ratio) per doubling in the prevalence of exposure.

## Discussion

We evaluated SNP-based genetic correlation and explored bidirectional causal association between depression and periodontitis. LDSC showed weak genetic correlation. LCV analysis provided estimates incompatible with genetic causation. Application of LHC-MR and CAUSE exploited genome wide data for exposure traits to test for bidirectional causation between depression and periodontitis, while accommodating correlated pleiotropy and maximizing statistical power. LHC-MR and CAUSE, along with four conventional MR methods, consistently lacked support for bidirectional causation between depression and periodontitis. Genetic correlation analysis indicated minimal shared genetic etiology, implying that phenotypic associations between depression and periodontitis are due to other mechanisms. Analysis of local genetic correlation could also not provide significant evidence for shared genetic influences. This might be owed to the fact that polygenicity of periodontitis is not yet fully discovered in the presently available GWAS. Point estimates, however, were compatible with putative causal effects.

Similar genome wide genetic correlation studies in periodontitis-associated phenotypes, including type 2 diabetes and glycemic traits, have failed to yield convincing evidence supporting a shared genetic link with depression (3, 50, 51). Our findings contrast with previous observational research suggesting bidirectional association between depression and periodontitis. However, results from observational studies have been inconsistent and controversy remains regarding the association between depression and periodontitis, in part due to variations in study design and appropriate statistical methods for providing unbiased associations. The largest meta-analysis on the relationship of periodontal disease with depression pooled estimates from 17 cross-sectional and eight case-control studies and reported summary ORs of 1.08 (95% CI: 0.88;1.32) and 1.70 (95% CI: 1.01;2.83), respectively (6). The meta-analysis showed high heterogeneity and the authors noted that most of the included studies were prone to risk of bias. Previous meta-analyses included fewer cross-sectional and case-control studies and showed no evidence for an association of depression and periodontitis (30, 52).

Most observational studies on the subject are cross-sectional or case-control. Cross-sectional studies reveal only undirected associations and thus reflect potential relationships in both directions; there is no way to separate them. Without longitudinal data and multiple measurements of exposure and outcome, we cannot hope to assess the direction (53). Only few prospective studies examined the association between depression and periodontitis. One prospective analysis of 720 participants of the 1982 Pelotas Birth Cohort applied the parametric g-formula to estimate the direct effect of depressive symptoms on periodontitis (7). The study adjusted for confounding factors and found a relative risk of 1.19 (95% CI: 1.04;1.36) for periodontitis. Notably, the follow-up period was only one year, which might have been too short to exclude the possibility for reverse causation. A longitudinal analysis using claims data from the Taiwan National Health Insurance Program compared 12,709 newly diagnosed periodontitis cases and 50,832 matched controls, and found a 76% higher risk (hazard ratio = 1.76; 95% CI: 1.53;1.89) for depression over a 10-year period, after regression-adjustment for confounding (54). A strength of the study is that start of follow-up was clearly defined by considering new cases to resemble an RCT and avoid time-related biases (55).

*In-vivo* studies have provided convincing causal mechanisms between periodontitis and depression. A potential mechanism connecting microbiota and neuroinflammation is *via* endotoxins induced by gram-negative bacteria (20). A recent pre-clinical *in-vivo* study found a possible direct invasion of *F. nucleatum* into brains of rats in which periodontitis and chronic stress was induced, suggesting a neuroinflammation caused by periodontal pathogen translocation through the blood-brain barrier (10). Another study showed induction of depression-like behavior in *P. gingivalis* treated mice and related these behavioral changes to higher levels of activated astrocytes, decreased brain-derived neurotropic factor and astrocytic p75 neurotrophic receptor in the hippocampus (56).

Our study has some limitations. First, the heritability of periodontitis was small with an estimate of 2%, which may bias the estimate of genetic correlation between depression and periodontitis. Nevertheless, this effect should be negligible, as the heritability of depression is substantially different from zero. Second, LCV produced a small genetic causality proportion. LCV models a latent factor which has a causal effect on both traits, and which mediates the genetic correlation between two traits. While LCV can detect reverse causation, it cannot estimate bidirectional causal relationships. Bidirectional causation would present as a close to null genetic causality proportion (32). Third, the number of instrumental SNPs for periodontitis was less than 10 in conventional MR analyses. However, the effects of these conventional MR methods are consistent with LHC-MR and CAUSE, which are less prone to weak instrument bias and have substantially higher statistical power. Fourth, conventional MR methods further assume that SNPs affect the outcome only through the exposure, and the use of binary exposures may violate this assumption. We prefer to treat these binary exposure estimates as a test of the null hypothesis as the interpretation of effect sizes from binary exposures may be subject to unrealistic assumptions related to the homogeneity of their effects (57). Last, analyses were restricted to European GWAS data and findings may be population-specific.

In summary, we triangulate evidence for a putative causal association between depression and periodontitis by applying several genetically informed methods. Although results do not suggest a causal connection between depression and periodontitis, we acknowledge the findings from *in-vitro* studies pointing to causal mechanisms. Accordingly, additional well-designed prospective studies that minimize observational study bias and replication efforts for our MR analyses with larger GWAS data are warranted. The future availability of more data with the power to explain a larger portion of the phenotypic variance could yet yield more evidence of a causal relationship between depression and periodontitis.

## Data availability statement

Depression summary data are available at https://datashare.ed.ac.uk/handle/10283/3203. Periodontitis summary data are available at https://data.bris.ac.uk/data/dataset/2j2rqgzedxlq02oqbb4vmycnc2.

## Ethics statement

The individual studies had previously obtained relevant ethical approval and participant consent. This study complied with all relevant ethical regulations, including the Declaration of Helsinki, and ethical approval for data collection and analysis was obtained by each study from local boards as described in the included GWAS.

## Author contributions

Conception and design: MN, BH, HB, SEB. Development of methodology: MN, HB, SEB. Acquisition of data (provided animals, acquired, and managed patients, provided facilities, etc.): MN, SEB. Analysis and interpretation of data (e.g., statistical analysis, biostatistics, computational analysis): MN, HB, SEB. Writing, review, and/or revision of the manuscript: MN, BH, TK, ZA, SR, BE, HB, SEB. Administrative, technical, or material support (i.e., reporting or organizing data, constructing databases): MN, BH, ZA, SR. All authors contributed to the article and approved the submitted version.

## Acknowledgments

The authors acknowledge and thank the investigators of the original GWAS studies for sharing summary data used in this study.

## Conflict of interest

The authors declare that the research was conducted in the absence of any commercial or financial relationships that could be construed as a potential conflict of interest.

## Publisher’s note

All claims expressed in this article are solely those of the authors and do not necessarily represent those of their affiliated organizations, or those of the publisher, the editors and the reviewers. Any product that may be evaluated in this article, or claim that may be made by its manufacturer, is not guaranteed or endorsed by the publisher.
