# Supplementary material for: No bidirectional relationship between depression and periodontitis: A genetic correlation and Mendelian randomization study
Source: Front Immunol. 2022 Jul 22;13:918404. doi: 10.3389/fimmu.2022.918404 (PMC9355660; doi:10.3389/fimmu.2022.918404)
Supplement: Supplementary file 1 [file DataSheet_1.docx]

**No bidirectional relationship between depression and periodontitis: a genetic correlation and Mendelian randomization study**

Michael Nolde et al.

Supplementary tables

Supplementary Table S1 Associations of single nucleotide polymorphisms for depression

|  |  |  |  | Estimates for depression | | | | Estimates for periodontitis | | |
| --- | --- | --- | --- | --- | --- | --- | --- | --- | --- | --- |
| SNP | EA | OA | EAF | BETA | SE | P | F | BETA | SE | P |
| rs1002656 | T | C | 0.703 | -0.027 | 0.004 | 3.7e-12 | 49.0 | -0.004 | 0.017 | 0.835 |
| rs10061069 | C | G | 0.221 | -0.028 | 0.004 | 8.2e-11 | 42.9 | -0.022 | 0.019 | 0.242 |
| rs10149470 | A | G | 0.487 | -0.027 | 0.004 | 3.7e-14 | 58.2 | 0.017 | 0.015 | 0.278 |
| rs1021363 | A | G | 0.355 | 0.030 | 0.004 | 4.4e-16 | 67.1 | 0.003 | 0.016 | 0.875 |
| rs1045430 | T | G | 0.479 | -0.025 | 0.004 | 7.3e-13 | 52.2 | -0.007 | 0.016 | 0.637 |
| rs10774600 | T | C | 0.166 | -0.027 | 0.005 | 3.4e-08 | 30.9 | -0.005 | 0.025 | 0.843 |
| rs10789214 | T | C | 0.566 | 0.019 | 0.004 | 4.4e-08 | 30.4 | 0.009 | 0.016 | 0.572 |
| rs10817969 | T | G | 0.717 | 0.026 | 0.004 | 3.1e-11 | 44.8 | -0.018 | 0.017 | 0.278 |
| rs10913112 | T | C | 0.377 | -0.026 | 0.004 | 3.4e-13 | 53.8 | 0.004 | 0.018 | 0.820 |
| rs1095626 | T | C | 0.580 | -0.026 | 0.004 | 7.1e-14 | 56.9 | 0.027 | 0.015 | 0.081 |
| rs11135349 | A | C | 0.471 | -0.029 | 0.004 | 6.0e-17 | 71.0 | -0.016 | 0.016 | 0.327 |
| rs113188507 | A | G | 0.284 | 0.022 | 0.004 | 1.9e-08 | 32.1 | 0.008 | 0.018 | 0.645 |
| rs1152578 | T | C | 0.436 | -0.022 | 0.004 | 6.4e-10 | 38.8 | 0.003 | 0.016 | 0.854 |
| rs11579246 | A | G | 0.907 | 0.038 | 0.006 | 5.7e-10 | 39.0 | 0.018 | 0.025 | 0.478 |
| rs115938232 | A | G | 0.939 | -0.057 | 0.007 | 2.6e-14 | 57.4 | 0.022 | 0.052 | 0.666 |
| rs12052908 | A | T | 0.532 | -0.022 | 0.004 | 4.4e-10 | 39.5 | -0.022 | 0.016 | 0.166 |
| rs1226412 | T | C | 0.792 | 0.026 | 0.004 | 3.5e-09 | 35.4 | 0.010 | 0.020 | 0.592 |
| rs12624433 | A | G | 0.258 | 0.023 | 0.004 | 7.4e-09 | 33.9 | 0.018 | 0.018 | 0.305 |
| rs12923444 | A | C | 0.562 | -0.021 | 0.004 | 1.3e-09 | 37.4 | -0.011 | 0.016 | 0.493 |
| rs12967143 | C | G | 0.698 | -0.031 | 0.004 | 3.7e-16 | 67.4 | 0.029 | 0.017 | 0.081 |
| rs12967855 | A | G | 0.330 | 0.026 | 0.004 | 1.2e-12 | 51.3 | 0.008 | 0.016 | 0.618 |
| rs13084037 | A | G | 0.774 | -0.024 | 0.004 | 7.1e-09 | 34.0 | -0.009 | 0.019 | 0.656 |
| rs1343605 | A | C | 0.384 | 0.031 | 0.004 | 6.2e-18 | 75.6 | 0.025 | 0.016 | 0.124 |
| rs1354115 | A | C | 0.624 | 0.021 | 0.004 | 7.1e-09 | 34.0 | 0.012 | 0.016 | 0.481 |
| rs1409379 | T | C | 0.764 | 0.025 | 0.004 | 1.7e-09 | 36.9 | 0.017 | 0.018 | 0.342 |
| rs141954845 | A | G | 0.388 | 0.023 | 0.004 | 8.1e-10 | 38.3 | 0.014 | 0.017 | 0.411 |
| rs143186028 | T | G | 0.178 | 0.028 | 0.005 | 2.3e-09 | 36.3 | 0.024 | 0.021 | 0.233 |
| rs1448938 | A | G | 0.417 | 0.021 | 0.004 | 1.3e-09 | 37.4 | 0.008 | 0.016 | 0.604 |
| rs1466887 | T | C | 0.551 | -0.020 | 0.004 | 4.1e-08 | 30.6 | -0.009 | 0.016 | 0.570 |
| rs1568452 | T | C | 0.385 | 0.025 | 0.004 | 8.1e-12 | 47.5 | -0.005 | 0.016 | 0.739 |
| rs16887442 | T | C | 0.435 | 0.020 | 0.004 | 8.6e-09 | 33.6 | 0.005 | 0.016 | 0.767 |
| rs169235 | A | G | 0.753 | -0.023 | 0.004 | 3.0e-08 | 31.2 | 0.016 | 0.018 | 0.349 |
| rs17641524 | T | C | 0.209 | -0.032 | 0.004 | 1.5e-13 | 55.4 | 0.038 | 0.020 | 0.055 |
| rs1890946 | T | C | 0.467 | -0.024 | 0.004 | 2.7e-11 | 45.1 | 0.022 | 0.016 | 0.156 |
| rs1933802 | C | G | 0.454 | -0.022 | 0.004 | 2.6e-10 | 40.6 | 0.005 | 0.016 | 0.746 |
| rs1956373 | T | G | 0.744 | -0.023 | 0.004 | 2.1e-08 | 31.9 | -0.014 | 0.017 | 0.410 |
| rs1982277 | T | C | 0.759 | 0.028 | 0.004 | 1.4e-11 | 46.3 | -0.003 | 0.018 | 0.867 |
| rs198457 | T | C | 0.192 | -0.029 | 0.005 | 3.0e-10 | 40.3 | -0.029 | 0.021 | 0.176 |
| rs200949 | A | G | 0.874 | 0.048 | 0.005 | 2.5e-19 | 82.0 | 0.000 | 0.023 | 0.996 |
| rs2029865 | A | T | 0.453 | -0.020 | 0.004 | 1.2e-08 | 33.0 | 0.016 | 0.016 | 0.307 |
| rs2043539 | A | G | 0.418 | 0.027 | 0.004 | 9.9e-15 | 60.8 | -0.015 | 0.016 | 0.347 |
| rs2187490 | T | G | 0.911 | -0.034 | 0.006 | 3.8e-08 | 30.7 | 0.004 | 0.030 | 0.885 |
| rs2247523 | C | G | 0.532 | -0.021 | 0.004 | 4.4e-09 | 35.0 | 0.007 | 0.015 | 0.647 |
| rs2509805 | T | C | 0.321 | 0.022 | 0.004 | 9.2e-09 | 33.5 | 0.014 | 0.018 | 0.429 |
| rs2568958 | A | G | 0.616 | 0.037 | 0.004 | 8.5e-25 | 107.3 | -0.006 | 0.016 | 0.710 |
| rs263645 | A | T | 0.544 | 0.022 | 0.004 | 3.7e-10 | 39.9 | 0.027 | 0.015 | 0.084 |
| rs2670139 | T | C | 0.761 | -0.027 | 0.004 | 1.2e-10 | 42.1 | 0.032 | 0.017 | 0.069 |
| rs2876520 | C | G | 0.527 | -0.023 | 0.004 | 2.3e-10 | 40.8 | -0.010 | 0.016 | 0.535 |
| rs301799 | T | C | 0.569 | -0.025 | 0.004 | 1.4e-12 | 51.0 | 0.024 | 0.016 | 0.122 |
| rs30266 | A | G | 0.330 | 0.031 | 0.004 | 1.4e-16 | 69.3 | -0.004 | 0.017 | 0.830 |
| rs3099439 | T | C | 0.529 | -0.028 | 0.004 | 5.0e-15 | 62.2 | -0.001 | 0.016 | 0.936 |
| rs3213572 | A | G | 0.474 | 0.022 | 0.004 | 7.6e-10 | 38.4 | 0.009 | 0.015 | 0.541 |
| rs33431 | T | C | 0.614 | 0.020 | 0.004 | 4.8e-08 | 30.2 | -0.005 | 0.016 | 0.737 |
| rs34488670 | T | C | 0.789 | -0.025 | 0.004 | 6.0e-09 | 34.3 | 0.014 | 0.019 | 0.454 |
| rs34937911 | T | C | 0.884 | 0.030 | 0.005 | 4.1e-08 | 30.5 | -0.024 | 0.025 | 0.320 |
| rs35553410 | T | C | 0.746 | -0.024 | 0.004 | 1.4e-09 | 37.2 | -0.003 | 0.018 | 0.876 |
| rs3793577 | A | G | 0.466 | -0.023 | 0.004 | 8.4e-11 | 42.8 | -0.015 | 0.015 | 0.341 |
| rs3823624 | T | C | 0.807 | 0.027 | 0.004 | 2.0e-09 | 36.5 | 0.036 | 0.021 | 0.079 |
| rs4346585 | T | C | 0.696 | -0.024 | 0.004 | 7.1e-10 | 38.6 | -0.006 | 0.018 | 0.745 |
| rs45510091 | A | G | 0.947 | 0.045 | 0.008 | 1.8e-08 | 31.4 | -0.007 | 0.038 | 0.857 |
| rs4772087 | T | C | 0.373 | 0.023 | 0.004 | 3.9e-10 | 39.8 | -0.027 | 0.017 | 0.110 |
| rs55943003 | A | G | 0.245 | 0.025 | 0.004 | 3.5e-09 | 35.4 | -0.018 | 0.020 | 0.357 |
| rs56314503 | T | G | 0.749 | -0.025 | 0.004 | 2.9e-10 | 40.3 | 0.004 | 0.018 | 0.806 |
| rs56887639 | A | G | 0.726 | -0.028 | 0.004 | 1.5e-12 | 50.8 | 0.013 | 0.017 | 0.459 |
| rs57344483 | A | G | 0.926 | -0.038 | 0.007 | 1.8e-08 | 31.2 | 0.018 | 0.028 | 0.526 |
| rs58104186 | A | G | 0.469 | 0.024 | 0.004 | 1.8e-11 | 45.9 | -0.004 | 0.016 | 0.813 |
| rs58621819 | A | T | 0.790 | -0.024 | 0.004 | 1.6e-08 | 32.5 | -0.059 | 0.020 | 0.003 |
| rs59283172 | A | G | 0.107 | -0.033 | 0.006 | 1.0e-08 | 33.3 | -0.035 | 0.026 | 0.176 |
| rs5995992 | T | C | 0.716 | -0.027 | 0.004 | 1.3e-11 | 46.5 | 0.014 | 0.017 | 0.400 |
| rs60157091 | T | C | 0.515 | 0.020 | 0.004 | 1.4e-08 | 32.7 | 0.016 | 0.016 | 0.313 |
| rs61902811 | A | G | 0.368 | -0.026 | 0.004 | 1.4e-12 | 51.0 | -0.026 | 0.016 | 0.114 |
| rs61990288 | A | G | 0.508 | -0.026 | 0.004 | 1.7e-13 | 55.2 | -0.041 | 0.015 | 0.008 |
| rs62188629 | A | G | 0.314 | 0.024 | 0.004 | 7.1e-10 | 38.6 | -0.004 | 0.017 | 0.809 |
| rs67436663 | C | G | 0.240 | -0.026 | 0.004 | 9.4e-10 | 38.0 | -0.025 | 0.019 | 0.188 |
| rs6783233 | T | C | 0.283 | 0.022 | 0.004 | 2.9e-08 | 31.2 | -0.008 | 0.018 | 0.657 |
| rs7030813 | T | C | 0.374 | 0.025 | 0.004 | 3.1e-12 | 49.4 | -0.009 | 0.016 | 0.575 |
| rs7117514 | A | G | 0.542 | -0.020 | 0.004 | 7.3e-09 | 34.0 | -0.008 | 0.016 | 0.614 |
| rs7193263 | A | G | 0.668 | -0.024 | 0.004 | 4.3e-10 | 39.6 | -0.035 | 0.016 | 0.033 |
| rs7198928 | T | C | 0.616 | 0.024 | 0.004 | 4.4e-11 | 44.1 | -0.013 | 0.016 | 0.412 |
| rs7200826 | T | C | 0.255 | 0.028 | 0.004 | 3.7e-12 | 49.0 | 0.010 | 0.018 | 0.577 |
| rs7227069 | A | G | 0.433 | 0.024 | 0.004 | 1.5e-11 | 46.2 | 0.017 | 0.016 | 0.296 |
| rs7241572 | A | G | 0.201 | 0.028 | 0.004 | 2.7e-10 | 40.5 | -0.013 | 0.020 | 0.519 |
| rs725616 | T | C | 0.364 | 0.020 | 0.004 | 1.9e-08 | 32.1 | 0.014 | 0.016 | 0.399 |
| rs72710803 | A | C | 0.912 | -0.041 | 0.006 | 5.3e-11 | 43.7 | -0.019 | 0.026 | 0.459 |
| rs75581564 | A | G | 0.116 | 0.030 | 0.005 | 3.2e-08 | 31.1 | 0.001 | 0.026 | 0.976 |
| rs7585722 | T | C | 0.846 | -0.027 | 0.005 | 2.7e-08 | 31.4 | 0.011 | 0.022 | 0.605 |
| rs7624336 | T | G | 0.209 | 0.024 | 0.004 | 4.0e-08 | 30.6 | -0.004 | 0.021 | 0.831 |
| rs7659414 | A | C | 0.578 | -0.020 | 0.004 | 1.2e-08 | 33.0 | -0.007 | 0.016 | 0.687 |
| rs7685686 | A | G | 0.575 | 0.020 | 0.004 | 2.6e-08 | 31.5 | -0.018 | 0.016 | 0.252 |
| rs7758630 | A | T | 0.405 | -0.022 | 0.004 | 5.6e-10 | 39.1 | 0.002 | 0.016 | 0.908 |
| rs7807677 | T | C | 0.550 | 0.024 | 0.004 | 1.8e-11 | 45.9 | -0.007 | 0.015 | 0.656 |
| rs78337797 | T | G | 0.878 | 0.031 | 0.005 | 3.4e-08 | 31.0 | -0.029 | 0.026 | 0.255 |
| rs7837935 | T | G | 0.152 | -0.029 | 0.005 | 3.3e-09 | 35.5 | -0.001 | 0.021 | 0.973 |
| rs7932640 | T | C | 0.442 | 0.028 | 0.004 | 1.6e-15 | 64.5 | -0.012 | 0.016 | 0.457 |
| rs8037355 | T | C | 0.556 | -0.023 | 0.004 | 3.9e-11 | 44.3 | -0.005 | 0.016 | 0.768 |
| rs913930 | A | G | 0.643 | -0.021 | 0.004 | 2.4e-08 | 31.6 | -0.002 | 0.016 | 0.894 |
| rs9363467 | T | C | 0.604 | 0.024 | 0.004 | 6.4e-11 | 43.3 | 0.034 | 0.016 | 0.032 |
| rs9545360 | A | C | 0.181 | -0.027 | 0.005 | 5.0e-09 | 34.7 | 0.012 | 0.021 | 0.554 |
| rs9592461 | A | G | 0.487 | 0.022 | 0.004 | 9.1e-10 | 38.1 | 0.012 | 0.016 | 0.436 |
| rs997934 | T | C | 0.380 | 0.020 | 0.004 | 4.8e-08 | 30.2 | 0.002 | 0.016 | 0.893 |

EA, effect allele. OA, other allele. EAF, effect allele frequency. SE, standard error.

Supplementary Table S2 Associations of single nucleotide polymorphisms for periodontitis

|  |  |  |  | Estimates for periodontitis | | | | Estimates for depression | | |
| --- | --- | --- | --- | --- | --- | --- | --- | --- | --- | --- |
| SNP | EA | OA | EAF | BETA | SE | P | F | BETA | SE | P |
| rs10143801 | A | G | 0.708 | -0.084 | 0.017 | 8.7e-07 | 24.1 | -0.001 | 0.005 | 0.893 |
| rs138868497 | T | C | 0.986 | 1.639 | 0.332 | 8.2e-07 | 24.3 | 0.034 | 0.026 | 0.205 |
| rs151226594 | T | G | 0.984 | -0.367 | 0.077 | 1.8e-06 | 22.8 | -0.002 | 0.017 | 0.928 |
| rs2921075 | C | G | 0.672 | 0.092 | 0.018 | 5.4e-07 | 25.1 | -0.007 | 0.005 | 0.152 |
| rs4811024 | C | G | 0.093 | -0.134 | 0.029 | 4.6e-06 | 21.0 | -0.008 | 0.007 | 0.297 |
| rs73155039 | A | G | 0.984 | 0.832 | 0.176 | 2.2e-06 | 22.4 | 0.005 | 0.019 | 0.812 |
| rs76734229 | A | G | 0.930 | -0.176 | 0.037 | 1.9e-06 | 22.7 | -0.006 | 0.008 | 0.434 |
| rs9954920 | T | C | 0.643 | 0.077 | 0.016 | 2.4e-06 | 22.3 | 0.015 | 0.004 | 0.001 |

EA, effect allele. OA, other allele. EAF, effect allele frequency. SE, standard error.

Supplementary Table S3 Heterogeneity of Wald ratios and MR-Egger test for directional pleiotropy

| Exposure | Heterogeneity | | |  |
| --- | --- | --- | --- | --- |
|  | Q | Degrees of Freedom | P | I² |
| Depression | 89.6 | 93 | 0.58 | 0 |
| Periodontitis | 13.2 | 7 | 0.07 | 0.40 |
|  | MR-Egger test for directional pleiotropy | | |  |
|  | Intercept | Standard error | P |  |
| Depression | 0.014 | 0.01 | 0.175 |  |
| Periodontitis | 0.003 | 0.004 | 0.53 |  |
